# Risk and protective factors for health behaviour in adolescence in Europe

**DOI:** 10.1038/s41598-023-45800-1

**Published:** 2023-10-30

**Authors:** Annamaria Zsakai, Fanny Zselyke Ratz-Sulyok, Beatrix Koronczai, Petra Varro, Erika Toth, Szilvia Szarvas, Tamas Tauber, Zsolt Karkus, Kinga Molnar

**Affiliations:** 1 Department of Biological Anthropology, Faculty of Science, ELTE, Eotvos Lorand University, Pazmany P. S. 1/C, Budapest, 1117 Hungary; 2 Health Promotion and Education Research Team, Hungarian Academy of Sciences, Budapest, Hungary; 3 Department of Developmental and Clinical Child Psychology, Faculty of Education and Psychology, ELTE, Eotvos Lorand University, Budapest, Hungary; 4 Department of Physiology and Neurobiology, Faculty of Science, ELTE, Eotvos Lorand University, Budapest, Hungary; 5 Department of Microbiology, Faculty of Science, ELTE, Eotvos Lorand University, Budapest, Hungary; 6 Veres Palne Gymnasium, Budapest, Hungary; 7 Apaczai Csere Janos Gymnasium, ELTE, Eotvos Lorand University, Budapest, Hungary; 8 Department of Anatomy, Cell and Developmental Biology, Faculty of Science, ELTE, Eotvos Lorand University, Budapest, Hungary

**Keywords:** Disease prevention, Health policy, Public health, Quality of life

## Abstract

The purpose of the analysis was to identify the risk and protective factors for health behaviour in European adolescents from population health status and expenditure, mental health status, sexual life, social life and education indices and the existence of national strategies, programmes. National and international databases providing information on the presumed health behaviour predictors were used in the analysis. The existence of national health strategies, the level of health expenditure, the socioeconomic conditions, the level of education and literacy had significant influence on the health-risk behaviour of adolescents in the European societies. Six clusters of European countries were extracted by considering the health behaviour risks and health protection strategies. National health strategies combined with governmental support for health prevention and action plans have the most effective impact on the health-risk behaviour of adolescents.

## Introduction

Public policy on health behaviour aims to give support for people to make healthy choices. Prevention programmes aim to minimize the burden of diseases and disease-associated risk factors, while health promotion programmes usually address behavioural risk factors and support people to increase control over their health^1^. Children and adolescents are among the main beneficiaries of prevention and health education programmes. However, one of the biggest challenges of health programmes promoting health behaviour to children and adolescents is not only to increase the level of knowledge of children exposed to a health education programme, but also to encourage them to change their lifestyle, to increase health awareness and to establish positive health behaviours^2^.

Recent studies have emphasized the need to develop online prevention, monitoring and support programs for young people^3^. The main risk factors for risky health behaviour are psychological factors (substance misuse, mental disorders, impulsivity, low self-esteem, poor social problem-solving, hopelessness), individual negative life events and family adversity (parental divorce or separation, child abuse, parental mental disorders, bullying and rejection), sociodemographic and educational factors (low socioeconomic status, restricted educational achievement, social isolation), chronic disease or disability^4, 5^.

The countries in Europe have developed their own prevention programmes and strategies to fight against risky health behaviour among children and adolescents in the last 2 decades. The aim of the analysis was to identify the main risk and protective factors for health behaviour in adolescence and young adulthood in Europe among population health status and expenditure, mental health status, sexual life, social life and education indices and the existence of national strategies, programmes. Health-related population indicators were used to identify types, groups of societies by the effectiveness of health promotion and prevention strategies.

Reducing health risky behaviour in children and adolescents requires a clear understanding of their main risk and protective factors. The overview of children and adolescent risk-taking behaviour statistics (facts are presented in the Results section) emphasize the importance of new strategies, models and discussions in Hungary and in the societies with similar effectiveness in health education, promotion and prevention. The analyses of the effectivity of former interventions, programmes could help to identify best-practice prevention models and to support unified actions and responses at national level.

## Subjects and methods

### Data sources

The databases, online sources used to collect statistics and data on health status and expenditures, mental health status, sexual and reproductive life, lifestyle and healthy behaviour, social life, education indices of European populations are presented in Table 1. The statistics and data were collected from the interval between 2014 and 2022 from 27 European countries (Belgium, Croatia, Czech Republic, Denmark, Estonia, Finland, France, Germany, Great Britain, Greece, Hungary, Ireland, Italy, Latvia, Lithuania, Norway, Poland, Portugal, Russia, Serbia, Slovakia, Slovenia, Spain, Sweden, Switzerland, The Netherlands, Ukraine). Data in the relationship analysis between health behaviour risks and health protection strategies were available for only 15 countries (Croatia, Czech Republic, Denmark, Estonia, Finland, Hungary, Ireland, Latvia, Lithuania, Norway, Portugal, Russia, Serbia, Sweden, Switzerland) (Supplementary information).

**Table 1:** Databases and reports used as data sources in the analysis^6–15^.

Data sources	Studied countries, examined period
Databases	
Eurostat	All the studied countries, 2014–2020
EU Kids Online 2020 survey*	19 studied countries, 2017–2019
Public Health, European Commission	All the studied countries, 2014–2020
Organisation for Economic Co-operation and Development	All the studied countries, 2014–2021
European Health Information Gateway	All the studied countries, 2014–2018
Reports	
EuroChild, UNICEF*	23 countries, 2020–2021
Global Health Observatory Data Repository*	21 countries, 2014–2019
Situation of child and adolescent health in Europe	All the studied countries, 2017
WHO Global eHealth survey 2015*	15 countries, 2015
Statista	All the studied countries, 2018

*Missing data from the referred databases were completed by collecting data from the national surveys and databases.

### Ethics declaration

Data for the analyses were collected from open access databases and publications. The protocol, methods and aims of the studies—those results were involved in the analyses—were approved by national and international ethical boards before acceptance for publication. All methods were carried out in accordance with relevant guidelines and regulations, informed consent was obtained from all subjects and/or their legal guardian(s). The protocol, methods and aims of the present study were approved by the Institutional Ethical Board of Faculty of Education and Psychology, Eotvos Lorand University (approval number: 2022/58) and the directors of the participated schools in 2022.

### Statistical analyses

Spearman's rank correlation analysis was used to study the pairwise relationship between the studied health status and expenditures, mental health status, sexual and reproductive life, lifestyle and healthy behaviour, social life, education indices in the studied 27 European countries. Hierarchical cluster analysis was used to generate clusters of countries using Ward’s method of measuring squared Euclidean distance by health behaviour risks (suicide prevalence, adolescent pregnancies, alcohol consumption, drug dependence, smoking, physical activity level) and health protection strategies. The Ward's method, one of the most frequently used methods in cluster analysis to minimise the within-cluster variance, starts with n clusters of size 1 and continues until decrease the number of clusters to keep the number of clusters as small as possible. The Euclidean distance was used for the presented analysis since it is also one of the most widely used methods in distance measure to estimate the countries’ relationship to the other countries and clusters of countries in the cluster analysis, for each countries the squared Euclidean distance to the cluster means was calculated. Statistical analysis was performed with SPSS v. 23. Hypotheses were tested at the level 5% random error.

## Results

### Relationship between the health risk behaviour indicators and the studied health status, health expenditures indices

The correlation analyses revealed that among the health status and expenditure indices the level of health expenditures, the number of general paediatricians had the highest impact on the health risk behaviour of children (Table 2). Among the studied health risk behaviour, the adolescent pregnancy rate was the most sensitive to the level of national health expenditures and health status indices, the higher level of national health expenditures, the higher rate of general paediatricians, the smaller rate of adolescent pregnancies was found. The rate of infant deaths increased by the rate of adolescent pregnancies. The rate of abortions in women aged under 20 years strongly correlated with adolescent pregnancy rate and the level of physical activity in children, the higher rate of abortions in adolescents and young women, the lower level of physical activity and the lower rate of adolescent pregnancies were found.

**Table 2:** Spearman's rank correlation coefficients between health risk behaviour indicators (columns) and the studied health and expenditure indices (rows; only the significant coefficients are presented, *p* < 0.05).

	Suicide-14	Suicide15-	Adol pregn	Alcohol	Drug	Smoking	Phys act	Leisure act
Health status and expenditure indices								
Total pharmaceutical expenditure^a^			− 0.595					− 0.480
Total health expenditure (GDP%)^a^	− 0.581	− 0.529	− 0.614			− 0.545		
Abortions/1000 live births under 20 years^a^			− 0.681				− 0.436	0.474
General paediatricians/100,000 inhabitants^a^			− 0.390			− 0.389		− 0.392
Incidence of cancer/100,000 inhabitants^a^				0.395				
Infant deaths/1000 live births^b^			0.753				− 0.471	
Adults reporting a chronic disease^a^								
15–24 years reporting a chronic disease^a^							− 0.557	0.636
Unmet health care app (16–19 years)^a^								
Expenditure of mental health care^a^							0.585	

*Suicide-14:* suicide and intentional self-harm, 0–14 years, per 100,000 population, *Suicide 15-:* suicide and intentional self-harm, 15–29 years, per 100,000 population, *Adol pregn:* adolescent pregnancy rate (per 1000 women), *Alcohol:* pure alcohol consumption, litres per capita, age 15–29 years, *Drug:* drug dependence and toxicomania, 15–29 years, per 100,000 population, *Smoking:* proportion of 15-year-old adolescents smoking daily, *Phys act:* performing health-enhancing aerobic and muscle-strengthening physical activity 15–19 years at least once a week, *Leisure act:* Percentage of children in households where at least one child does not participate in a “regular leisure activity” and/or "go on holiday away from home at least one week per year.

data sources (see Table 1).

^a^WHO Global eHealth survey 2015.

^b^European Health Information Gateway.

### Relationship between the health risk behaviour indicators and mental health status indicators

The rate of very good self-perceived health status in youths aged between 16 and 19 years negatively correlated with the level of suicide and intentional self-harm in children, the rate of drug dependence in youths aged 15–29 years and the proportion of 15-year-old smoking children, but positively correlated with the level of physical activity in children (Table 3). Physical activity level showed also positive correlation with rate of young people with high life satisfaction.

**Table 3:** Spearman's rank correlation coefficients between health risk behaviour indicators (columns) and the studied mental health indicators (rows; only the significant coefficients are presented, *p* < 0.05).

	Suicide-14	Suicide15-	Adol pregn	Alcohol	Drug	Smoking	Phys act	Leisure act
Mental health status indices								
Mental disorders/100,000 inhabitants^a^								
Rate of very good self-perceived health (16–19 years)^b^	− 0.392				− 0.410	− 0.542	0.427	
Young people (%, 13–15 years) with high-life satisfaction^c^							0.423	

*Suicide -14:* suicide and intentional self-harm, 0–14 years, per 100,000 population, *Suicide 15-:* suicide and intentional self-harm, 15–29 years, per 100,000 population, *Adol pregn:* adolescent pregnancy rate (per 1000 women), *Alcohol:* pure alcohol consumption, litres per capita, age 15–29 years, *Drug:* drug dependence and toxicomania, 15–29 years, per 100,000 population, *Smoking:* proportion of 15-year-old adolescents smoking daily, *Phys act:* performing health-enhancing aerobic and muscle-strengthening physical activity 15–19 years at least once a week, *Leisure act:* Percentage of children in households where at least one child does not participate in a “regular leisure activity” and/or "go on holiday away from home at least one week per year.

data sources (see Table 1).

^a^European Health Information Gateway.

^b^Public Health, European Commission.

^c^Statista.

### Relationship between the health risk behaviour indicators and sexual life, reproductive life indices

Mothers' age at first birth also revealed significant relations with the health risk behaviours of children: the younger mean age at first birth, the higher rate of suicide and intentional self-harm in children and the higher rate of smoking children were found (Table 4).

**Table 4:** Spearman's rank correlation coefficients between health risk behaviour indicators (columns) and the studied sexual life indicators (rows; only the significant coefficients are presented, *p* < 0.05).

	Suicide-14	Suicide 15-	Adol pregn	Alcohol	Drug	Smoking	Phys act	Leisure act
Sexual life indices								
Mothers' age at first birth (the studied children’s mothers)^a^	− 0.546	− 0.528				− 0.534		
People (%) 15 years used a condom at last intercourse^b^								
Seeing sexual images online in past year (15–16 years)^b^								
Adolescent pregnancy rate/1000 women^b^						0.417		

*Suicide-14:* suicide and intentional self-harm, 0–14 years, per 100,000 population, *Suicide 15-:* suicide and intentional self-harm, 15–29 years, per 100,000 population, *Adol pregn:* adolescent pregnancy rate (per 1000 women), *Alcohol:* pure alcohol consumption, litres per capita, age 15–29 years, *Drug:* drug dependence and toxicomania, 15–29 years, per 100,000 population, *Smoking:* proportion of 15-year-old adolescents smoking daily, *Phys act:* performing health-enhancing aerobic and muscle-strengthening physical activity 15–19 years at least once a week, *Leisure act:* Percentage of children in households where at least one child does not participate in a “regular leisure activity” and/or "go on holiday away from home at least one week per year.

data sources (see Table 1).

^a^Eurostat.

^b^WHO Global eHealth survey 2015.

Condom use among adolescents and seeing sexual images online did not show significant relationship with the adolescents’ health risk behaviour in the studied countries.

### Relationship between the health risk behaviour indicators and social life indices

By considering the social indices it could be stated that the rate of divorce, the ratio of children bullied in school and the level of children in institutional care were in positive correlation with the level of suicide and intentional self-harm in children in the studied European countries. By considering the level of smoking children, the higher divorce rate, the lower level of kindness among children in school, the higher level of children bullied in school, the higher level of excessive internet use in children and the higher level of children living in institutional care, the higher level of daily smoking was found in children. The higher level of child poverty, the lower level of kindness in school among children related with lower level of physical activity in children (Table 5).

**Table 5:** Spearman's rank correlation coefficients between health risk behaviour indicators (columns) and the studied social life indices (rows; only the significant coefficients are presented, *p* < 0.05).

	Suicide -14	Suicide 15-	Adol pregn	Alcohol	Drug	Smoking	Phys act	Leisure act
Social life indices								
Children at risk of poverty/social exclusion^a^							− 0.397	0.714
Crude divorce rate^a^	0.523	0.590				0.435		
Children (%, 11, 13 and 15 years) agree their class is kind^b^						− 0.398	0.433	− 0.381
Children (%, 11, 13, 15 years) are bullied in school^b^	0.696	0.608		0.405		0.489		
Children (%) at risk of poverty or social exclusion^b^								0.611
Children (%) excessive internet use^b^						0.419		
Negative online experiences in past year (15–16 years)^b^								
Children (%) in institutional care/100,000 inhabitants^c^	0.431	0.659				0.535		

*Suicide -14:* suicide and intentional self-harm, 0–14 years, per 100,000 population, *Suicide 15-:* suicide and intentional self-harm, 15–29 years, per 100,000 population, *Adol pregn:* adolescent pregnancy rate (per 1000 women), *Alcohol:* pure alcohol consumption, litres per capita, age 15–29 years, *Drug:* drug dependence and toxicomania, 15–29 years, per 100,000 population, *Smoking:* proportion of 15-year-old adolescents smoking daily, *Phys act:* performing health-enhancing aerobic and muscle-strengthening physical activity 15–19 years at least once a week, *Leisure act:* Percentage of children in households where at least one child does not participate in a “regular leisure activity” and/or "go on holiday away from home at least one week per year.

data sources (see Table 1).

^a^Eurostat.

^b^WHO Global eHealth survey 2015.

^c^EuroChild.

### Relationship between the health risk behaviour indicators and education indices

Education indices showed significant relations with the level of suicide and intentional self-harm and the level of physical activity in children, namely, underachievement rate in school increased the prevalence of the suicidal feelings in both age-groups, while the higher level of young adults having only primary education and the lower level of literacy rate increased the prevalence of suicide and intentional self-harm in children. These two education indices showed correlation with the level of physical activity in children, too (Table 6).

**Table 6:** Spearman's rank correlation coefficients between health risk behaviour indicators (columns) and the studied education indices (rows; only the significant coefficients are presented, *p* < 0.05).

	Suicide -14	Suicide 15-	Adol pregn	Alcohol	Drug	Smoking	Phys act	Leisure act
Education indices								
Children (%) to teachers and academic staff^a^								
Early leavers (%) from education^a^								
Population (%) primary education only (25 years)^a^		0.467						0.469
Literacy rate (%, 15 years)^a^		− 0.500						− 0.442
Underachievement rate in school^b^	0.395	0.427						

*Suicide -14:* suicide and intentional self-harm, 0–14 years, per 100,000 population, *Suicide 15-:* suicide and intentional self-harm, 15–29 years, per 100,000 population, *Adol pregn:* adolescent pregnancy rate (per 1000 women), *Alcohol:* pure alcohol consumption, litres per capita, age 15–29 years, *Drug:* drug dependence and toxicomania, 15–29 years, per 100,000 population, *Smoking:* proportion of 15-year-old adolescents smoking daily, *Phys act:* performing health-enhancing aerobic and muscle-strengthening physical activity 15–19 years at least once a week, *Leisure act:* Percentage of children in households where at least one child does not participate in a “regular leisure activity” and/or "go on holiday away from home at least one week per year.

data sources (see Table 1).

^a^Organisation for Economic Co-operation and Development.

^b^EU Kids Online 2020 survey.

### Relationship between the health risk behaviour indicators and the existence of national health strategies and programmes

Having national strategies and programmes in the countries revealed significant correlations with the indices of health risk behaviour in children. Having national strategy on health promoting and policy of having sexuality education decreased the level of adolescent pregnancies, while having education programmes on sexual partner violence and funding of eHealth programmes from public sources decreased the prevalence of suicide and intentional self-harm in children. Funding of eHealth programmes from public sources also increased the level of physical activity in children, and having a budget allocated for health prevention strategies related with the lower level of drug dependence and toxicomania in children (Table 7).

**Table 7:** Spearman's rank correlation coefficients between health risk behaviour indicators (columns) and the studied national health strategy indicators (rows; only the significant coefficients are presented, *p* < 0.05).

	Suicide -14	Suicide 15-	Adol pregn	Alcohol	Drug	Smoking	Phys act	Leisure act
National strategies, programmes								
National strategy on health promoting^a^			− 0.469					
Policy of having sexuality education^b^			− 0.412					
Education addresses sexual partner violence^c^	− 0.451	− 0.449						
Funding of eHealth programmes (public sources)^c^	− 0.422						0.439	
Having a budget allocated for these strategies^c^					0.533			

*Suicide-14:* suicide and intentional self-harm, 0–14 years, per 100,000 population, *Suicide 15-:* suicide and intentional self-harm, 15–29 years, per 100,000 population, *Adol pregn:* adolescent pregnancy rate (per 1000 women), *Alcohol:* pure alcohol consumption, litres per capita, age 15–29 years, *Drug:* drug dependence and toxicomania, 15–29 years, per 100,000 population, *Smoking:* proportion of 15-year-old adolescents smoking daily, *Phys act:* performing health-enhancing aerobic and muscle-strengthening physical activity 15–19 years at least once a week, *Leisure act:* Percentage of children in households where at least one child does not participate in a “regular leisure activity” and/or "go on holiday away from home at least one week per year.

data sources (see Table 1).

^a^Situation of child and adolescent health in Europe.

^b^Global Health Observatory Data Repository.

^c^WHO Global eHealth survey 2015.

### European countries clustering by children’s health behaviour risks and health protection strategies

**Figure 1:**
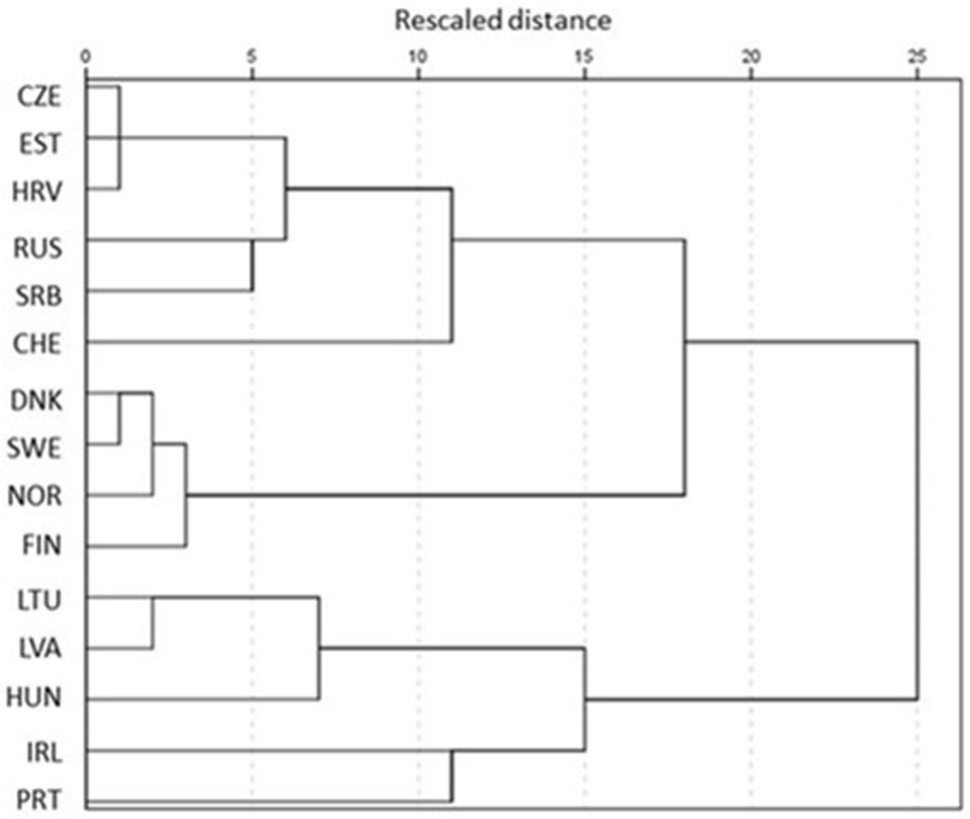
Dendrogram of the studied European countries by the health behaviour risks (suicide prevalence, adolescent pregnancies, alcohol consumption, drug dependence, smoking, physical activity level) and health protection strategies (National strategies, programmes items in Table 2; not all the studied countries had data for these factors).

By considering the health behaviour risks (suicide prevalence, adolescent pregnancies, alcohol consumption, drug dependence, smoking, physical activity level) and health protection strategies in the studied countries (data were available for only 15 countries, WHO Global eHealth Survey 2015)^11^ 6 clusters of countries were extracted (Fig. 1, Table 8). Denmark, Sweden, Norway and Finland formed the first cluster (‘Nordic’ cluster), they had national strategies on health promoting and sexual education in schools, their countries could allocate a budget for these strategies and had remarkable funding contribution for eHealth programmes provided by both public sources and public–private partnership sources. These health investments resulted in very low level of adolescent pregnancies, low level of alcohol consumption in youths, low level of suicide and intentional self-harm in adolescents under the age of 15 and a very high ratio adolescents doing physical activity regularly. The level of suicide and intentional self-harm in older adolescents, proportion of adolescents smoking daily, the ratio of adolescents with drug dependence were also very low in these countries except for Finland, where these health behavioural indicators were worser than in the other members of the cluster (Table 8).

**Table 8:** Clusters of countries by the health behaviour risks and health protection strategies (not all the 27 studied countries had data for health strategies in the WHO Global eHealth Survey 2015)—the clusters are ordered by considering the national health strategies and education programmes in the cluster countries.

Country	Nat strat prom	Nat strat health	Budget strat	Sex ed	Ed sex viol	Health pub-priv	Health pub	Suicide -14	Suicide 15-	Adol pregn	Alcohol	Drug	Smok	Phys act	Leisure act
DNK	+	+	+	P+S	+	Low	V high	0.1	7	3	10	0.3	14	42	19
SWE	−	+	+	P+S	+	Low	V high	0.3	10	4	7	0.1	13	45	27
NOR	+	+	+	P+S	+	No	V high	0.2	12	4	6	0.1	9	46	16
FIN	+	+	+	P+S	+	Low	V high	0.3	16	6	8	1.1	20	42	23
CZE	+	+	−	P+S	no	Low	Low	0.5	10	12	13	0.1	22	21	33
EST	+	+	+	P+S	+	No	V high	1.0	12	11	16	0.4	22	18	29
HRV	−	+	+	P+S	+	No	V high	0.5	8	10	10	0.1	27	21	30
CHE	+	−	−	P+S	+	Low	Low	0.4	7	3	9	0.1	19	13	9
LTU	+	−	−	−	−	Low	High	1.0	18	13	13	0.2	34	33	46
LVA	+	+	+	P+S	+	No	Low	0.7	14	15	11	0.2	32	23	46
HUN	−	+	−	P+S	+	No	V high	0.1	7	25	11	0.1	26	28	59
IRL	+	+	−	P+S	+	No	Low	0.2	11	7	11	4.4	12	36	55
PRT	+	+	−	P+S	+	No	High	0.1	4	8	10	0.1	11	12	62
RUS	+	+	+	S	−	Low	High	0.6	17	22	8	1.0	19	12	25
SRB	−	+	−	−	+	No	Low	0.5	10	15	7	0.2	10	13	30

*Nat strat prom:* national strategy on health promoting schools, *Sex ed:* policy of having sex education in schools, *ED sex viol:* country undertakes age- and gender-appropriate education on sexual partner violence, *Health pub-priv:* the proportion of funding contribution for eHealth programmes provided by public–private partnership funding sources over the previous 2 years, *Health pub:* the proportion of funding contribution for eHealth programmes provided by public funding sources over the previous 2 years, *Nat strat health:* the country had a national strategy for child and adolescent health and development that has been adopted within the last 5 years, *Budget strat:* having a budget allocated for these strategies, *Suicide -14:* suicide and intentional self-harm, 0–14 years, per 100,000 population, *Suicide 15-:* suicide and intentional self-harm, 15–29 years, per 100,000 population, *Adol pregn:* adolescent pregnancy rate (per 1000 women), *Alcohol:* pure alcohol consumption, litres per capita, age 15–29 years, *Drug:* drug dependence and toxicomania, 15–29 years, per 100,000 population, *Smok:* proportion of 15-year-old adolescents smoking daily, *Phys act:* performing health-enhancing aerobic and muscle-strengthening physical activity 15–19 years at least once a week, *Leisure act:* Percentage of adolescents in households where at least one child does not participate in a “regular leisure activity” and/or "go on holiday away from home at least one week per year.

P + S: in primary and secondary schools, P: only in primary schools, S: only in secondary schools, v high: very high.

The second cluster was formed by the Czech Republic, Estonia and Croatia (East-Central European countries I cluster). The health promotion and education strategies were not as strong and frequent as in the Nordic cluster countries, but still stronger than in the other clusters (had sex education both in primary and secondary education systems, had national strategies for health and sex education in schools, but the funding contribution for eHealth programmes was lower both from the public and private sectors). The level of adolescent pregnancies, the level of smoking and alcohol consumption in adolescents were higher, while the level of physical activity in adolescents was lower than in the cluster of countries from the Scandinavian region (Table 8).

Switzerland formed a cluster (‘Swiss’ cluster) by having health and sex education programmes in the schools, but not having budget for these programmes. The health behaviour metrics were very good in this country except for the low level of physical activity in adolescents.

In the clusters of Lithuania, Latvia and Hungary (East-Central European countries II cluster) and Ireland and Portugal (Western European cluster) the countries had mixed types of national health strategies and health education programmes, had worse health behaviour metrics than in the Nordic, East-Central European and Swiss clusters. These two clusters were very close not only by considering their health behaviour metrics (Table 8) but also in the dendrogram (Fig. 1). The East-Central European countries III cluster consisted of Russian and Serbia, where the health behaviour metrics were similar to the countries’ metrics in East-Central European countries I cluster (these 2 clusters were close to each other in the dendrogram)—with the exception of higher level of adolescent pregnancies and higher level of suicide and intentional self-harm among adolescents and young adults. The missing of health and sex education programmes and strategies in Russian and Serbia might explain these differences between the 2 clusters.

## Discussion

The relationship among health risk behavioural indicators and health status, mental health, sexual life, reproductive life, lifestyle, education indicators were analysed by relying on data from European societies in the last 10 years. It could be stated that the existence of national health strategies, the level of health expenditure, the poverty in the micro- or macro-environment, the level of education and literacy had significant influence on the health-risk behaviour of adolescents in the European societies. There is some evidence that besides these factors health risk behaviour in childhood is determined by genetic factors, family history, national history, and many other factors^16, 17^. Bozzini and his colleagues reviewed the most important factors associated with risk behaviours in adolescence, and emphasized that risk behaviour studies focused mostly on the social, environmental, school, neighborhood, and family factors, while the physical and psychological traits of individual adolescents has not been explored yet^18^. Keel and Keiser emphasized the importance of nursing interventions to increase health protection among adolescents, nurses can support adolescents to encourage the decision to avoid health risk behaviour^19^. The research results of Tsitsimpikou and her colleagues revealed that health problems among adolescents were the main reason to consider changing lifestyle and health behaviour habits, although adolescents were aware of that health-risk behaviours were harmful. This led light on the importance of effective health risk education in schools^20^.

Health prevention strategies are intentions taken at national and international levels to prevent the onset or the complications of manifested diseases, injuries, impaired health statuses by targeting healthy and not healthy individuals. Health prevention programmes aim to serve guidelines on risk reduction to individuals and groups of individuals. The results of the present analysis highlight the importance of appropriate prevention and education strategies, especially for children and adolescents living in high-risk groups (in poverty, institutional care). Individual health decisions of adolescents are not made in social isolation but rather in interaction with others, in early stages with family members and experts, and in a later age-interval with a bigger influence of friends. Tsitsimpikou et al. drew similar conclusions by highlighting that health preventive youth strategies should target negative peer influences in adolescence^20^. Winter et al. revealed that adolescents with low levels of self-control and low level of socioeconomic background were vulnerable to developing multiple health risk behaviours in a prospective cohort study of Dutch adolescents and suggested enhancing self-control for preventing multiple health risk behaviours in the health education and preventive strategies^21^. The existence of national health strategies combined with significant health expenditures and budget allocated for health prevention and action plans revealed to be the most effective impact on the health-risk behaviour of adolescents and young adults. Nowadays the arsenal of eHealth services (digital technologies) and mHealth services (mobile technology) support health prevention programmes. Online, electronic education materials serve as effective training tools for health education.

The results of the presented analyses are in accordance with some of the results of the HBSC (Health Behaviour in School-aged Children) surveys. The HBSC surveys regularly measure the health and health behaviours of adolescents through cross-national investigation from 1983 in more than 40 countries all over the world. The HBSC cross-country comparisons revealed for example that countries can be divided into subgroups by the tendencies of adolescent mental health problems (studied in the Nordic European countries)^22^. However, HBSC surveys produced by interdisciplinary teams in countries and regions, cross-country evidence reviews usually not concentrate on the main aim of the presented analysis, i.e. how the health risk behavioural indicators and health status, mental health, sexual life, reproductive life, lifestyle, education indicators are related in the European countries. The results of the presented analyses are supposed to give further details and tones to the determinants, the risk and protective factors for health behaviour in adolescence that topic has been enormously studied by several research groups including the ‘HBSC family’.

## Conclusions

The main aim of the presented analysis was to reveal similarities and differences among the European societies, to identify groups of societies by considering the health risk behaviour indicators, the studied health, social, education indices and national health strategy indicators. The Health Promotion and Education Research Team’s (formed by the authors in Eotvos Lorand University and the Hungarian Academy of Sciences, Budapest, Hungary) task to contribute to the Hungarian national prevention strategies by not only by constructing materials for the health preventive education, but also by introducing effective examples of health prevention strategies in the European countries.

The analysis of the relationship analyses between the health risk behaviour indicators and the studied health status, health expenditures, mental health status, sexual life, reproductive life, lifestyle, healthy behaviour, social life, education indices revealed that the existence of national health strategies, the level of health expenditure, the socioeconomic conditions, the level of education and literacy had significant influence on the health-risk behaviour of adolescents in the European societies. 6 clusters of European countries were extracted by considering the health behaviour risks and health protection strategies among Belgium, Croatia, Czech Republic, Denmark, Estonia, Finland, France, Germany, Great Britain, Greece, Hungary, Ireland, Italy, Latvia, Lithuania, The Netherlands, Norway, Poland, Portugal, Russia, Serbia, Slovakia, Slovenia, Spain, Sweden, Switzerland, Ukraine.

The Health Promotion and Education Research Team aims to provide an online platform to access educational resources for supporting school-aged children’s health behaviour development. The modules of the educational package will introduce the most important health-related facts by considering the age characteristics and knowledge of adolescents (aged 9–16 years) and will contain interactive tests to support their sufficient learning. In December 2021, the development of the toolkit has started by collecting data on the knowledge and attitude of adolescents toward healthy lifestyle and environmental awareness, as well as on their lifestyle habits via an online questionnaire. The first module (Sex education) of the electronic package has been available since January 2023 (https://tuti.elte.hu)^23^. The planned electronic education material will help the prevention work among Hungarian children and adolescents not only in health education but also in environment and media awareness education.

### Supplementary Information

Supplementary Information.

## Data Availability

The dataset (data.sav) generated and/or analysed during the current study are available in the https://egyk.elte.hu/sites/default/files/data.xlsx repository.

## Abbreviations

BEL: Belgium
CHE: Switzerland
CZE: Czech Republic
DEU: Germany
DNK: Denmark
ESP: Spain
EST: Estonia
FIN: Finland
FRA: France
GBR: Great Britain
GRC: Greece
HRV: Croatia
HUN: Hungary
IRL: Ireland
ITA: Italy
LTU: Lithuania
LVA: Latvia
NLD: The Netherlands
NOR: Norway
POL: Poland
PRT: Portugal
RUS: Russia
SRB: Serbia
SVK: Slovakia
SVN: Slovenia
SWE: Sweden
UKR: Ukraine
